# Automatic Quantification of Anterior Lamina Cribrosa Structures in Optical Coherence Tomography Using a Two-Stage CNN Framework

**DOI:** 10.3390/s21165383

**Published:** 2021-08-09

**Authors:** Md Habibur Rahman, Hyeon Woo Jeong, Na Rae Kim, Dae Yu Kim

**Affiliations:** 1 Department of Electrical and Computer Engineering, Inha University, Incheon 22212, Korea; habibur21@inha.edu (M.H.R.); qhsh9713@gmail.com (H.W.J.); 2 Department of Ophthalmology, Inha University, Incheon 22212, Korea; 3 Inha Research Institute for Aerospace Medicine, Inha University, Incheon 22212, Korea; 4 Center for Sensor Systems, Inha University, Incheon 22212, Korea

**Keywords:** optical coherence tomography, lamina cribrosa, Bruch’s membrane opening, convolutional neural network

## Abstract

In this study, we propose a new intelligent system to automatically quantify the morphological parameters of the lamina cribrosa (LC) of the optical coherence tomography (OCT), including depth, curve depth, and curve index from OCT images. The proposed system consisted of a two-stage deep learning (DL) model, which was composed of the detection and the segmentation models as well as a quantification process with a post-processing scheme. The models were used to solve the class imbalance problem and obtain Bruch’s membrane opening (BMO) as well as anterior LC information. The detection model was implemented by using YOLOv3 to acquire the BMO and LC position information. The Attention U-Net segmentation model is used to compute accurate locations of the BMO and LC curve information. In addition, post-processing is applied using polynomial regression to attain the anterior LC curve boundary information. Finally, the numerical values of morphological parameters are quantified from BMO and LC curve information using an image processing algorithm. The average precision values in the detection performances of BMO and LC information were 99.92% and 99.18%, respectively, which is very accurate. A highly correlated performance of R^2^ = 0.96 between the predicted and ground-truth values was obtained, which was very close to 1 and satisfied the quantification results. The proposed system was performed accurately by fully automatic quantification of BMO and LC morphological parameters using a DL model.

## 1. Introduction

Glaucoma is an optic nerve disease in which damage to retinal ganglion cells (RGCs) and axons progresses, owing to the structural damage of the optic nerve head (ONH), thereby leading to visual field loss [1]. The lamina cribrosa (LC) is located inside of the ONH and is made of a mesh-like porous structure of collagen fibers. The LC is damaged and deformed in the ONH structure during the progression of glaucoma [2,3]. Therefore, the morphological deformation of the LC could be related to the progression of glaucoma. It has been reported that the thickness of the LC is thinner in glaucoma patients than in normal individuals [4,5]. Modern imaging technologies such as enhanced depth imaging, spectral-domain optical coherence tomography (SD-OCT) [6,7,8] and swept-source (SS)-OCT [9] enable the in vivo visualization of the LC and deep structures in the ONH. By using these optical technologies, LC changes in glaucoma eyes, such as thinning [5], focal defects [10], and posterior displacement [11], have been previously studied. In addition, the significance of LC changes on the progression of glaucoma was studied by the authors of [12,13], who reported that LC imaging is clinically relevant for glaucoma monitoring. However, it has previously been conveyed that there are some drawbacks, such as it being tedious, time-consuming, and costly, as well as producing errors due to the manual measurements and identification of the LC morphology changes [14,15]. Therefore, it was required to implement a system for automatically quantifying the changes in morphology due to glaucoma progression in the OCT images.

Many researchers have been studying how LC responds to changes and causes of eye diseases [16,17]. However, all the methods for the quantification of the LC structure are still conventional, traditional, and manual. To overcome mentioned limitations, recently, deep learning (DL)-based methods have attracted attention for analyzing the unstructured OCT images by measuring the deformation of LC fully automatically and more accurately. A DL-based image segmentation and motion correction was proposed in the previous work [18], where, to gain effective segmentation output in conjunctiva images with its motion blur, the process was done by the Attention-UNet convolutional neural network (CNN) method. A deep CNN-based method [19] was used to automatic detection of the Bruch’s membrane opening (BMO) in the SD-OCT volumes, where they did not pay attention to the LC investigation after BMO detection. Reference [20] proposed a custom DL algorithm to digitally stain tissue in OCT images in the ONH and made a comparison of its segmentation performance with the manual process. The previous study developed an automated OCT segmentation method by using a fully convolutional network via the application of the DenseNets with a post-processing mechanism [21]. In the study [22], the authors developed a U-net DL-Shortest path algorithm-based automated processing system for retinal segmentation, where U-net is a convolutional neural network architecture used for accurate and fast image segmentation [23]. George, Y. et al. [24] proposed an end-to-end attention-guided 3D DL model for detecting glaucoma which could estimate the visual function from the retinal shape. During training, the model improved the performance of identifying the finer ocular structures in OCT images. OCT image processing based on the delineation of LC from a 3D OCT image via a fully automated algorithm to parameterize the appearance of the LC and determine the difference in the glaucoma state was developed by Syga P. et al. [25]. In the previous study [26], an OCT noise reduction algorithm was applied that trained without noise-free ground-truth images by utilizing the DL method; the authors performed the segmentation process after manually cropping a portion of the OCT images. To extract the retinal nerve fiber layer, RGC inner layer and RGC complex area, Raja H. et al. [27] proposed a hybrid CNN model. In the study [28], the authors proposed an automated segmentation of the retinal layer by using customary operation. Authors in the former work [29] described a CNN model to detect the glaucoma in OCT images and used testing with concept activation vectors (TCAVs) to assume what image concepts CNN model utilize to generate prediction. For underlying high-level concepts trained by the CAV, TCAVs use directional derivatives to measure model prediction’s susceptibility where CAV provides an explanation of a neural net’s internal situation with regard to the human-friendly concepts [30].

From the above discussion, most researchers have developed DL-based systems in single-stage mode and focused on segmentation for glaucoma determination in OCT images. In this study, our intention is to construct an intelligent DL-based system for segmenting and extracting LC information from OCT images by comparing its structural condition in both glaucoma and healthy eyes. Herein, we propose a two-stage DL model that enables the system to be fully automatic. The proposed DL model consists of detection and segmentation models. The detection model was implemented by using the YOLOv3 algorithm, where Darknet-53 was used as the backbone network to detect the BMO and LC region accurately. Moreover, we used the Attention U-Net model for the segmentation model to acquire the exact BMO points and LC curve information. In addition, post-processing involving polynomial regression to attain anterior LC curve boundary information is applied. Finally, the numerical values of the LC parameters are quantified by applying an image processing algorithm. The proposed system works in automatic mode and quantifies the BMO and LC more effectively than conventional methods and single-stage DL systems.

The rest of the paper is organized as follows. We explain the proposed method architecture and DL network in Section 2. In Section 3, we describe and explain the outcomes of testing the proposed system. Finally, we discuss and conclude the experimental results with a comparison of normal and glaucoma eyes in Section 4.

## 2. Methods

### 2.1. Dataset Preparation and its Information

**Figure 1:**
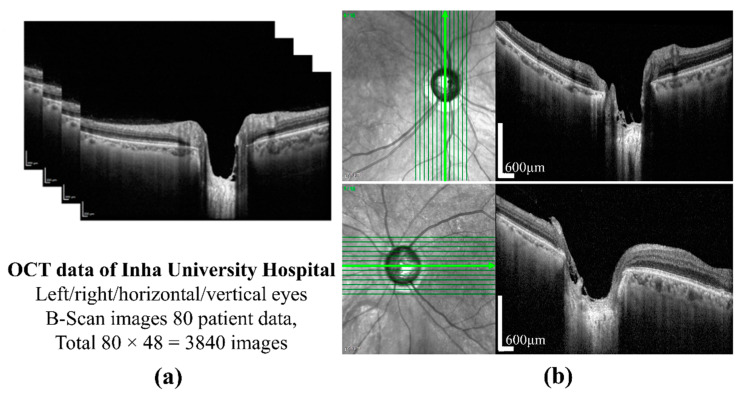
(**a**) OCT images from the Inha University Hospital; (**b**) an image was obtained by sampling near the optic nerve nipple. Scale bar: 600 µm.

The preparation of a perfect dataset is very important for DL-based methods. In this study, SD-OCT (Heidelberg Engineering GmbH, Heidelberg, Germany) images of 80 patients of different ages were provided by the Inha University Hospital. The OCT images of one patient are shown in Figure 1a. All procedures in the study conformed to the declaration and were approved by the institutional review board of the Inha University Hospital. The SD-OCT image size was 500 × 760 pixels, and cross-sectional vertical and horizontal images of the left and right eyes were provided by B-Scanning. Twelve images for each condition were selected, which provides a total of 3840 images. A cross-sectional image showing the sample of ONH is shown in Figure 1b. A labeled image collected from patients is essential for each model in supervised learning. First, the dataset for the object detection model was constructed by the sampling of 600 images from SD-OCT 2840 images, which were collected from 60 patients, the images from which were excluded from the patient data for testing purposes. To label the images for training, validation, and testing of the detection model, in this study, we used the LabelImg tool [31] to uniformly label each BMO and LC area on the OCT images and an XML file corresponding to the images generated to store the labeling information for the subsequent network training. The actual labeling mechanism is as follows: (i) the target area (BMO and LC area) was selected by a computer mouse, which was then (ii) double-clicked to mark it as the corresponding class, and finally (iii) “Save” was clicked once completed.

Each image in the training dataset has central coordinates $\left(X_{c},\;Y_{c},W,\;H\right)$ and the object class sequence number. However, the XML file takes out all target classes in that file and the corresponding coordinate values contain $\left(X_{min},\;Y_{min},X_{max},Y_{max}\right)$ in the upper left and lower corners, and $\left(X_{min}^{\prime },Y_{min}^{\prime },\;X_{max}^{\prime \prime },Y_{max}^{\prime \prime }\right)$ is the image scaling factor, which is obtained from the multiplied coordinate data.

Therefore, Equation (1) is used to convert the coordinates into the form of the central coordinate points. In the image label, the grid confidence degree is 1, and the coordinates of the center points are calculated as follows:

(1) $$\{\begin{array}{c} X_{c}=\frac{X_{min}^{\prime }+X_{max}^{\prime \prime }}{2},\;\;\;W=X_{max}^{\prime \prime }-X_{min}^{\prime }\\ Y_{c}=\frac{Y_{min}^{\prime }+Y_{max}^{\prime \prime }}{2},\;\;\;W=Y_{max}^{\prime \prime }-Y_{min}^{\prime } \end{array}$$

To obtain the robustness of the network, we saved and stored the training dataset as a file to the database by adding an appropriate label path file.

A bounding box defined the location information of the BMO and LC area, which was created by the object detection model during the generation of the labeled images. The output of the detection model was used as the input dataset for the semantic segmentation model. The detection area in the form of the bounding box was used in the making of the training dataset for the detection model. For validation testing of the dataset for the model, around 20% of the training data was configured, and optimal hyperparameters were obtained in the experiments as follows. 

On the other hand, 200 data items were used to select from the object detection dataset for the semantic segmentation model. To obtain the optimal hyperparameters, 20% of the data was used for verification purposes.

### 2.2. An Overview of the Proposed Method

**Figure 2:**
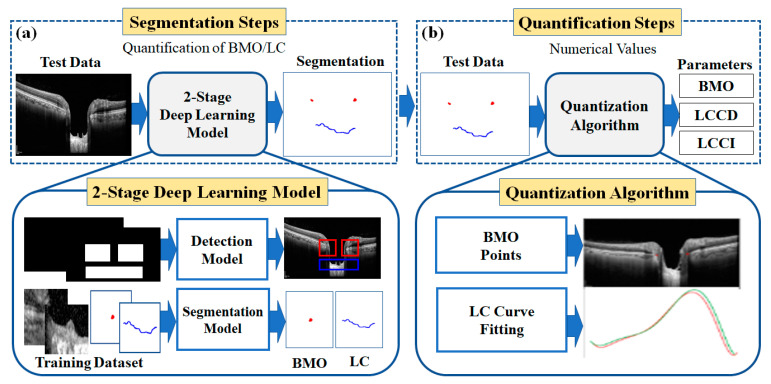
Overview of the proposed two-stage DL methods to quantify the BMO and LC morphological features: (**a**) segmentation steps consist of detection and a segmentation model for getting BMO and LC information; (**b**) quantification steps (LC depth (*LCD*), LC curve (*LCCD*), and LC curvature index (*LCCI*) numerical values calculation by image processing quantization algorithm from BMO and LC information).

The proposed method is applied to quantify the morphological features of the anterior LC in SD-OCT images. Figure 2 shows the overall configuration block diagram of the proposed two-stage DL quantification method step by step. We prepared the two datasets consisting of OCT images of normal and glaucoma eyes for training the DL model: one for object detection and one for the segmentation model. The generated training datasets were divided into three portions (one each for training, verification, and testing) to take advantage of them appropriately for each purpose of the system. The method was constructed as two steps: segmentation and quantification. The segmentation step consists of two DL models: detection and segmentation. The detection model was employed to detect the BMO and LC areas, while the detailed semantic division was performed by the segmentation model in the detected areas identified by the detection model. In the quantification step, the location information of the BMO and the anterior LC curve surface was acquired from the output of the segmentation model.

The quantification step involves post-processing and image processing algorithms to acquire morphological parameters from the numerical information. Post-processing was performed using polynomial regression. Finally, three types of morphological information were obtained: LC depth (*LCD*), LC curve depth (*LCCD*), and LC curvature index (*LCCI*). 

### 2.3. Descriptions of the Measurement Parameters

**Figure 3:**
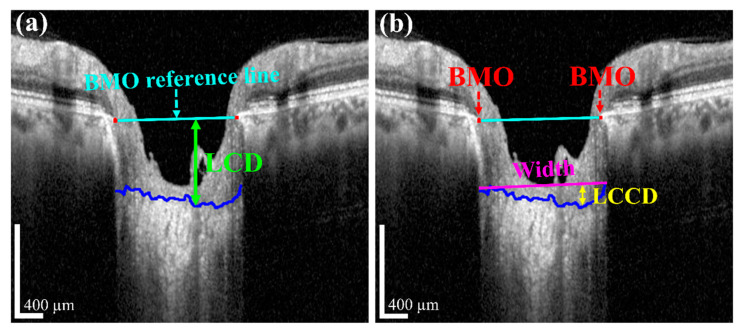
Measurement of *LCD* and *LCCD*: (**a**) *LCD* (lime green line) is the maximum distance from the BMO reference line (cyan line) to the anterior LC surface (blue curve); (**b**) *LCCD* (yellow line) is the maximum depth of the LC surface width (magenta line) to the anterior LC surface. Scale bar: 400 µm.

The measurement of two of the parameters for the proposed method, *LCD* and *LCCD* is exhibited in Figure 3. In Figure 3, the two red dots and blue jagged line are defined as BMO points and the anterior lamina cribrosa (LC) surface curve, respectively. As the goal of our proposed system was to measure or quantify the morphological features *LCD*, *LCCD*, and *LCCI*, the segmentation of BMO points and the LC curve is very essential. Therefore, in the proposed system, we segmented those two BMO points and the LC curve by segmentation process for quantifying the LC morphological features. In Figure 3a, the connection line of two BMO points is called the BMO reference line. The distance from the BMO reference line and the LC curve surface is defined as *LCD*. To measure the *LCCD*, a new reference line (the LC surface reference line) was set by connecting the two points on the anterior LC surface that meet with the lines drawn from each Bruch’s membrane termination points perpendicular to the BMO reference line. The length of this reference line was defined as the width (*W*) of the anterior LC curve to the surface.

After that, the *LCCD* was determined as the maximum depth from this width to the anterior LC surface, as shown in Figure 3b. *LCCI* [11] is calculated by dividing *LCCD* by the W (the anterior LC surface reference line), as follows (2):

(2) $$\mathrm{LCCI}=\frac{LCCD}{W}\times 100$$

### 2.4. Data Configuration Techniques

#### 2.4.1. Data Augmentation Technique

Image augmentation is a technique that can be used to artificially expand the size of a dataset by creating the modified versions of images in the prepared dataset. It is applied to prevent the overfitting error when training the DL model or a robust learning process. In this study, the augmentation technique was used to consider the changes in image data for prediction in the object detection and semantic segmentation processes in different ways. First, it works very effectively by considering the robustness of the model against various geometric changes that can occur during the collection of image data from a patient. In addition, random rotation, movement, and zoom transformation were applied to the input images to train the model. Second, gaudy noise and motion noise were added to mimic the noise generated while acquiring the images. Finally, vessel shadows were randomly added and then learned to take the patient’s blood vessels into account.

#### 2.4.2. Vessel Shadow Augmentation Technique

**Figure 4:**
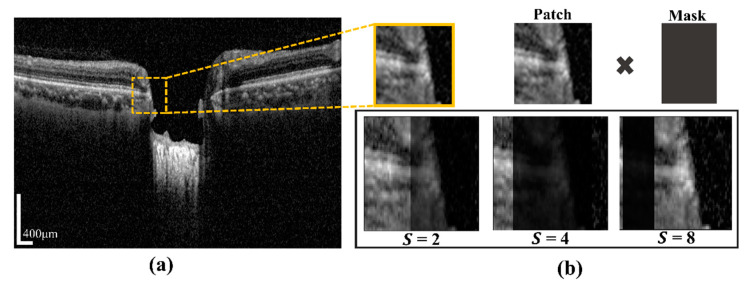
(**a**) The vascular shadow generation technique, (**b**) results according to shadow factor value (*S* = 2, 4, 8). Scale bar: 400 µm.

A certain part of the images becomes blurry due to blood vessel shadows (BVSs), which causes errors when inferring the semantic segmentation model. BVSs impact the performance of the model by lowering the intensity value of a rectangular shaped image. To address these limitations, in this study, we proposed Algorithm 1. In the proposed system, the novelty of the algorithm is to utilize the augmentation of input training patch images for minimizing prediction effects of BMO points and the LC curve with the ground-truth. This operation was carried by multiplying the mask shadow factor, which created a mask size smaller than the image patches. In addition, it was used to make the system robust in a test environment and in models with a large capacity. Even if the probability distribution of training and testing was changed by augmentation, the model with a large capacity was used to learn the representation of the test without affecting the existing prediction accuracy. The proposed technique was expressed as the following: by considering the input training sample image data *Xs*, the hyperparameter range of the shadow factor *S*, and the size of shadow width *W″*, the output of the augmented image *Xau* and the function of vessel shadow augmentation are contained in training sample image data *Xs.* After calculating the shadow width and height, the input image is multiplied with a shadow mask. Figure 4a shows a BVS randomly generated in the image by using S (which designates the degree of BVS and is specified in an arbitrary range of the BVS) and a mask made with an arbitrary width in the image. The generated BVS image after applying S is shown in Figure 4b.
**Algorithm 1.** Vessel Shadow Augmentation1: **input:** Training image Xs
2: **hyperparameters:** Range of shadow factor S, size of shadow width W″

3: **output:** Augment image Xau

4: Function vessel shadow augmentation (Xs)

5: Shadow width range $\left(W^{\prime \prime },Xs_{width}-W^{\prime \prime }\right)$

6: Shadow height $Xs_{height}$

7: Shadow mask Shadow width, Shadow height)
8: Shadow mask (Shadow mask × 1/S)9: Xs × shadow mask10: **end**

### 2.5. The Proposed Two-Stage DL Model

**Figure 5:**
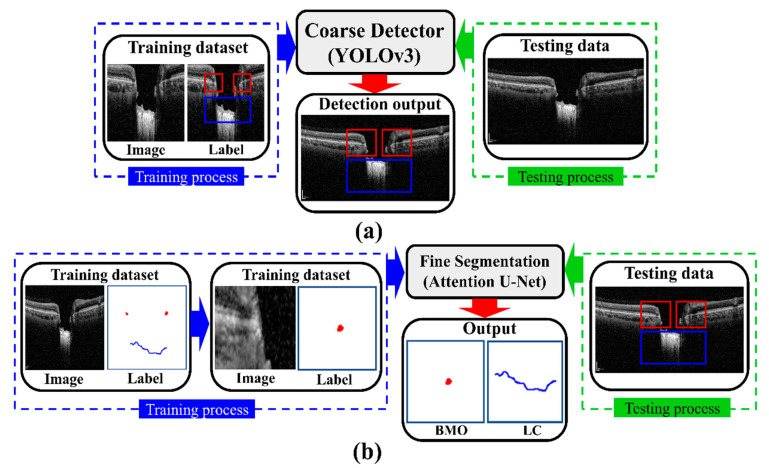
Block diagram of the proposed two-stage DL models: (**a**) the detection model with training and testing process, where the direction of blue, red, and green arrows indicate the working mechanism; (**b**) the segmentation model with training and testing process, where the direction of blue, red, and green arrows indicate the working mechanism.

The proposed DL model consists of two stages: an object detection model consisting of Coarse Detector YOLOv3 algorithms and semantic segmentation consisting of an Attention U-Net model. Figure 5 shows a block diagram of the proposed two-stage DL model. In the proposed two-stage model, we trained the two models sequentially. First, we trained the detection model for getting the exact location of the BMO and LC area within the OCT images and then observed the loss function for the YOLOv3 train model using our detection dataset, which is defined by Equation (4). Second, the detection results of the OCT image patches were used as the training dataset for the segmentation model, where the loss function in Equation (10) was calculated for the Attention U-Net model separately.

#### 2.5.1. The Coarse Object Detection Model

To detect the BMO and LC area on the OCT image, the coarse object detector plays an important role in roughly obtaining the target area to acquire information for quantification of *LCD*, *LCCD*, and *LCCI*. For training the detection model, a dataset with detection in the form of a bounding box was constructed, as discussed in Section 2.1. The selected images constitute the final training dataset through the data augmentation technique discussed in Section 2.4. For the detection mechanism, training of the data proceeded by using the YOLOv3 based Darknet-53 model [32], where Darknet-53 works as the backbone for the YOLOv3 network. For the regression problem, we considered object detection using the YOLOv3 method. It directly predicts the class probabilities and bounding box offsets from full input images with a single feed-forward CNN model. The YOLOv3 method divides the input image into grid cells of size J×J, whereby a grid cell is used to detect an object on the image when the center of the object falls within the grid cell.

The positional information for the bounding boxes is predicted by each grid cell and used to calculate the objectness score of the bounding box as follows:

(3) $$M_{i}^{j}=Q_{i,j}\left(Object\right)\times IOU_{predicted}^{groundtruth}$$

where $M_{i}^{j}$ is the objectness score of the bounding box in the grid cell and $Q_{i,j}\left(Object\right)$ represents the object function. The intersection over union (IOU) between the predicted and ground-truth boxes is defined as $IOU_{predicted}^{groundtruth}$. For the class predictions during training, the YOLO method was used independent logistic classifiers and binary cross-entropy function. In addition, it uses a multilabel approach, which allows classes to be more specific and be combined for individual bounding boxes of objects. Meanwhile, in terms of multilabel classification, binary cross-entropy is applied, instead of the SoftMax function. The proposed YOLOv3 method uses the binary cross-entropy of the predicted objectness score and ground-truth objectness score as loss functions defined as follows [33]:

(4) $$Z=\overset{G^{2}}{\underset{i=0}{\sum }}\overset{B}{\underset{j=0}{\sum }}U_{ij}^{object}\left[r_{i}^{j}\log\left({r^{\prime }}_{i}^{j}\right)-\left(1-r_{i}^{j}\right)\log\left(1-{r^{\prime }}_{i}^{j}\right)\right]$$

where $G^{2}$ is the number of grid cells, B is the number of bounding boxes, ${r^{\prime }}_{i}^{j}$ is the predicted objectness score, and $r_{i}^{j}$ is the ground-truth objectness score. 

The four predictions: $\left(v_{x},v_{y},\;v_{w},v_{h}\right)$ are defined positions in each bounding box. Based on this assumption, $(g_{x},g_{y}$) is the offset of the grid cell from the top corner of the image. From the top left corner of the image by $(b_{x},b_{y}$), the center positions of the final predicted bounding box is the offset, which is calculated as follows:

(5) $$\{\begin{array}{c} b_{x}=\sigma \left(v_{x}\right)+g_{x}\\ b_{y}=\sigma \left(v_{y}\right)+g_{y} \end{array}$$

where σ() is a sigmoid function. The width and height $(n_{w},n_{h}$) of the predicted box are respectively computed as:

(6) $$\{\begin{array}{c} n_{w}=p_{w}e^{v_{w}}\\ n_{h}=p_{h}e^{v_{h}} \end{array}$$

where $p_{w}$ and $p_{h}$ are the prior width and height of the bounding box calculated by the dimensional clustering. The ground-truth box consists of $k_{x},k_{y},\;k_{w},k_{h}$ according to the predicted parameters of $b_{x},b_{y},\;b_{w},b_{h}$, respectively. Thus, the ground-truth values of ${v^{\prime }}_{x},\;{v^{\prime }}_{y},\;{v^{\prime }}_{w},\;{v^{\prime }}_{h}$ can be calculated based on (5) and (6) as follows:

(7) $$\{\begin{array}{c} \sigma \left({v^{\prime }}_{x}\right)=k_{x}-g_{x}\\ \sigma \left({v^{\prime }}_{y}\right)=k_{y}-g_{y}\\ {v^{\prime }}_{x}=\log\left(k_{w}/p_{w}\right)\\ {v^{\prime }}_{h}=\log\left(k_{h}/p_{h}\right) \end{array}$$

During the training process for the YOLOv3 model, the image data were divided into 4 batches, while learning was performed for 300 epochs using the Adam optimizer function [34] with a learning rate of 0.0001. In Section 2.1, we mentioned that 20% of image data is used for validation to obtain the optimal hyperparameters for the proposed model. During the training period, when the loss function of the verification dataset did not decrease after 10 epochs repeated using the *ReduceLROnPlateau* (reduce learning rate on the plateau) strategy, the learning rate was reduced by 1/10. To complete the training, when the loss function of the validation dataset did not decrease after repeating for 30 epochs, we used the early stopping strategy mechanism.

#### 2.5.2. The Fine Segmentation Model

**Figure 6:**
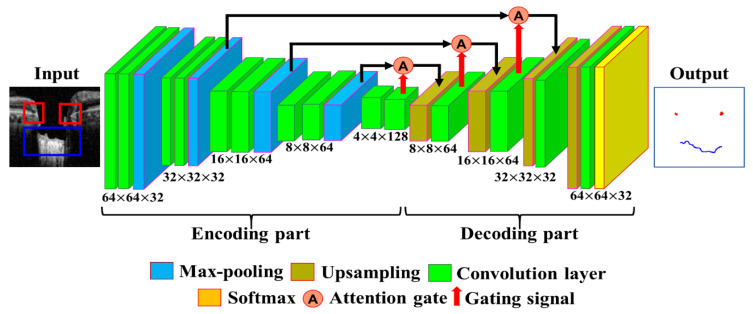
The proposed Attention U-Net model used for fine segmentation learning.

The segmentation model is defined by calculating the location information of BMO points and the LC curve on the output image of the object detection model. Data from 200 images out of 600 images were selected for training the coarse detector. During the training of the model, the image dataset was increased by dividing the BMO and LC area into patch units with a size of 64 × 64 pixels using the data augmentation technique described in Section 2.4. The segmentation process was trained with a total of 300,000 image patches. The architecture of the proposed Attention U-Net model used for the fine segmentation process is shown in Figure 6. For the training of the model, 300,000 image patches were divided into 16 batches, and learning over 200 epochs was performed using the Adam optimization function with a learning rate of 0.000005.

For validation purposes, 20% of the image dataset was used to obtain the optimal hyperparameter values from the training model. During the period of training, when the loss function of the verification dataset did not decrease after 15 epochs repeated using the *ReduceLROnPlateau* strategy, the learning rate was reduced by 1/10. To complete the training, when the loss function of the validation dataset did not decrease after repeating for 40 epochs, we used the early stopping strategy mechanism.

A loss function, which is the sum of the weights of focal loss [35] and generalized dice loss (GDL) [36], was designed and applied to solve the class imbalance problem. Focal loss works by balancing classes in the field of object detection and is computed as follows:

(8) $$Focal\;loss\left(P_{t}\right)={\left(1-P_{t}\right)}^{\gamma }\log\left(P_{t}\right)$$

where $P_{t}$ is the probability value obtained from the predicted value of the model, γ is used as an exponent to adjust the loss function for each class. The overall loss function is identical in form to the cross-entropy function. When the focal loss is used, a class with a high probability is given a small value, and one with a low probability is given a high loss function value to balance the imbalance problem. Moreover, the dice loss function is adopted by determining the resemblance of the overlapping area of the predicted and ground-truth regions in the model. The *GDL* function for multiple classes for training the model is computed as:

(9) $$GDL=1-2\times \frac{\sum_{l=1}^{c}w_{l}\sum_{n}r_{ln}p_{ln}}{\sum_{l=1}^{c}w_{l}\sum_{n}r_{ln}+p_{ln}}$$

where *c* is the number of classes, $w_{l}$ is the weighted value to provide invariance to different label set properties. r,p is defined as the precision (PR) and recall (RE), which is obtained by calculating the dice loss value for each class and multiplying the given weight values. The proposed loss function solves the class imbalance problem by summing the weighted values of these two functions. Computation of the final loss function is achieved by using:

(10) Final loss=α×Focal loss+β×GDL

For training the model, the values of α and β are both set to 1 and followed by simple addition where the focal loss and *GDL* loss are taken from (8) and (9), respectively.

### 2.6. The Post-Treatment and Quantification Method

For the quantification process, post-processing was applied to the erosion segment of the map information obtained by the proposed DL model. The anterior LC curve surface was calculated by applying a polynomial regression boundary on the map information. After the post-processing, the BMO, and anterior LC surface information were quantified by the image processing algorithm to obtain the final morphological features: *LCD*, *LCCD*, and *LCCI.*

#### Post-Processing Using Polynomial Regression and Quantifying the Morphological Information

**Figure 7:**
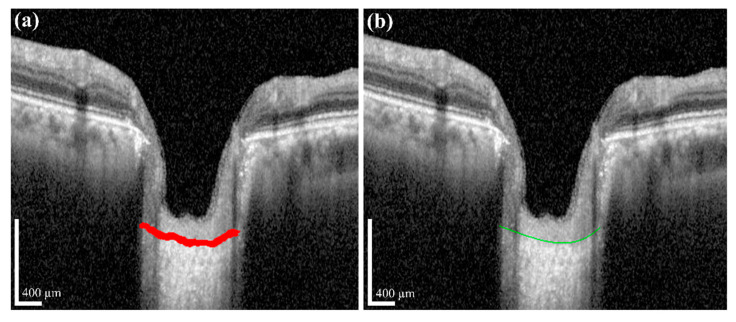
(**a**) Prediction of the anterior LC curve surface (red curve); (**b**) the result after applying the post-processing operation (green curve). Scale bar: 400 µm.

Post-processing was performed by applying a polynomial regression curve that estimates the curve model of the anterior LC boundary based on the acquired BMO and LC information from the semantic segmentation step. The learning data for the polynomial regression curve comprised LC boundary position information. Moreover, ridge regulation was defined as the N-order curve and the regression curve. The purpose of ridge regulation prevented the overfitting problem of the proposed model. Figure 7 shows the acquired anterior LC curve surface, in which Figure 7a presents the predicted LC surface model and Figure 7b presents the results of the image after applying the post-processing operation. After post-processing, we quantified the morphological information, such as *LCD*, *LCCD*, and *LCCI*, by the acquired BMO as well as the anterior LC curve surface. 

We applied image processing algorithms for numerical value computation to measure that information. In the image processing algorithm, we first took the original OCT image for the investigation. After that, the proposed DL model and post-processing were performed to obtain the boundary curve of the BMO points and the anterior LC surface. Subsequently, the two BMO points were connected to create a BMO reference line used to quantify the *LCD* information. To obtain accurate anterior LC curve information, a vertical dotted line was connected from two BMO points with the two ends of the anterior LC curve. The connection line between the ends of the anterior LC curve was defined as the width of the LC surface.

Based on this information, the *LCCD* was calculated by the deepest perpendicular distance from the width to the anterior LC surface. The distance for each depth was calculated by counting the number of pixels. The calculated pixel length was converted into a physical distance according to the image scale bar. From the obtained *LCCD* and width information, it was possible to calculate the *LCCI* according to the definition given in Section 2.3.

## 3. Experiment and Results

### 3.1. Experimental Platform

In this article, the experiments were performed on the Ubuntu 16.04 LTS system (Canonical Ltd., London, UK). The program was written in Python 3.6 under the development of PyCharm. Under the Darknet framework, YOLOv3-DL was run. The processor was an Intel Xeon (R) Silver 4112 CPU, and a NVIDIA graphic card was used to accelerate training.

### 3.2. Deep Learning Model Performance Evaluation

#### 3.2.1. Coarse Detection Performance Analysis

The performance of the object detection model was evaluated by a confusion matrix-based index. A comparison between the ground-truth and predicted values was created based on the confusion matrix mechanism. When the ground-truth and predicted values of the model are True then it is called True Positive (TP). When the ground-truth value is True, but the predicted value is False, then it is called True Negative (TN). False Positive (FP) is when both the ground-truth predicted values of the model are False. On the other hand, when the ground-truth value is False, but the predicted value is True, then it is called False Negative (FN). In this study, the detection operation of the BMO and LC area was performed based on the above four indicators.

**Figure 8:**
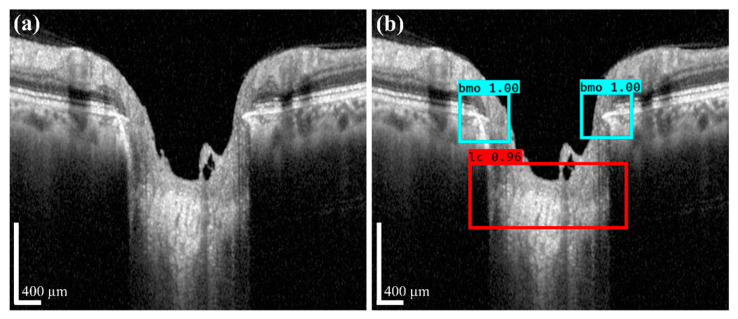
(**a**) An image used for learning the coarse detector performance; (**b**) the prediction results for the corresponding image (the cyan boxes represent the BMO detection area, and the red box represents the LC detection area). Scale bar: 400 µm.

**Figure 9:**
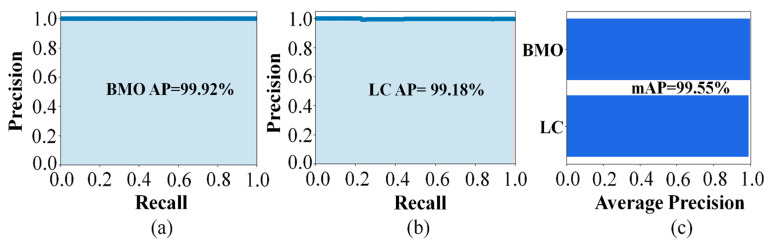
Learning results for the performance of the object detection model: Recall vs. Precision resulting in (**a**) 99.92% average precision (AP) for the BMO; (**b**) 99.18% (AP) for the LC; (**c**) the mean AP (mAP) for BMO and LC was 99.55%.

Assuming that P is the actual number of samples among target predictions, this is called precision. Equation (11) defines precision and recall, where precision and recall are the trade-off relation, and R is denoted as recall. Precision and recall are useful measures of prediction success when the categories are very imbalanced. In information recovery, precision is a measure of result relevancy, while recall measures how many truly relevant results are returned. The precision-recall results show a trade-off relationship for the different threshold values, and performance of the model varies according to the threshold value, which affects the precision and recall. The precision-recall curve is obtained by evaluating the performance by varying the confidence threshold value, which is a measure of how well the model selects objects for each object class. It is meaningful to evaluate precision-recall curves because an object detector with good performance obtains high values for both precision and recall:

(11) $$\{\begin{array}{c} Precision=\frac{TP}{TP+FP}\\ Recall=\frac{TP}{TP+FN} \end{array}$$

(12) $$mAP=\frac{\sum AP}{N_{classes}}$$

where *AP* is the average precision defined for all recalls obtained by changing the threshold value and can be measured by calculating the area under the precision–reproducibility curve, and *mAP* is the mean *AP.* If there are multiple objects, after calculating the *AP* for each object, it is summed and divided by the number of objects in (12). The detection results for the image are shown in Figure 8, in which Figure 8a,b depicts the original input image and the corresponding prediction results for the original image, respectively. Figure 9 shows the performance of the multi-class objects detection performance according to the prepared dataset, where the *AP* for the BMO classes was 99.92% and for the LC class was 99.18%. The mAP of detection accuracy for the BMO and LC was obtained as 99.55%.

#### 3.2.2. Fine Segmentation Performance Analysis

The performance evaluation of the semantic segmentation model depends on the two main information parameters, BMO, and LC. The evaluation index is based on measuring the distance between the BMO/anterior LC curve and the ground-truth information. Distance determination was evaluated based on two methods, such as: L1 (Manhattan) and L2 (Euclidean) distances. The distance between two points, such as (x^1^, y^1^) and (x^2^, y^2^), measured along x- and y-axes at right angles is called L1, which is expressed as: L1 = dx + dy, where dx = (x^1^ − x^2^) and dy = (y^1^ − y^2^). In contrast, the straight-line distance between two points (x^1^, y^1^) and (x^2^, y^2^) is called L2, which is expressed as: L2 = $\sqrt{{\left(dx\right)}^{2}+{\left(dy\right)}^{2}}$. Table 1 reports the calculated L1 and L2 distances. For the BMO, the mean and standard L1 distance values were 39.23 and 40.54 μm, and the mean and standard L2 distance values were 31.46 and 30.95 μm, respectively.

The calculation of the anterior LC curve was evaluated by using the difference between the average distance and the Hausdorff distance [37]. The average distance was calculated by the measurement between the prediction curve and the corresponding value of the ground-truth curve. When the distance was slightly different among most of the values, the distance between the two curves was different. Moreover, if there is a very large difference in a specific area, then the difference between the average distances cannot properly be evaluated. In these cases, the evaluation was conducted by the additional Hausdorff distance. This was an indicator of how far the two curves deviated from each other. In this way, the proposed system calculated the distance between each point of the curves. At this time, the minimum value among the distances between the points of the curve was compared with the point on the reference curve, and the Hausdorff distance was obtained by selecting the maximum value among the obtained minimum values. Table 2 reports the predicted results for the anterior LC curve by the segmentation model. However, the segmentation model confusion matrix scores for the BMO class were used to calculate the performance measures as follows: (13), (14), and (15). Accuracy was defined as the ratio with the sum of *TP* and *TN* and the sum of *TP*, *FP*, *FN*, and *TN*. The F1 score was defined as the harmonic mean of *RE* and *PR* [38], where *RE* and *PR* is the recall and precision, respectively.

(13) $$Accuracy=\frac{TP+TN}{TP+FP+FN+TN}$$

(14) $$F1\;Score=2\times \frac{\left(RE\times PR\right)}{\left(RE+PR\right)}$$

(15) $$Dice\;Coefficient=\frac{2\times TP}{\left(2\times TP+FP+FN\right)}$$

Table 3 reports the BMO class processing results from the segmentation algorithm and its measurement and evaluation values.

#### 3.2.3. Post-Processing and Quantification Performance Evaluation

**Figure 10:**
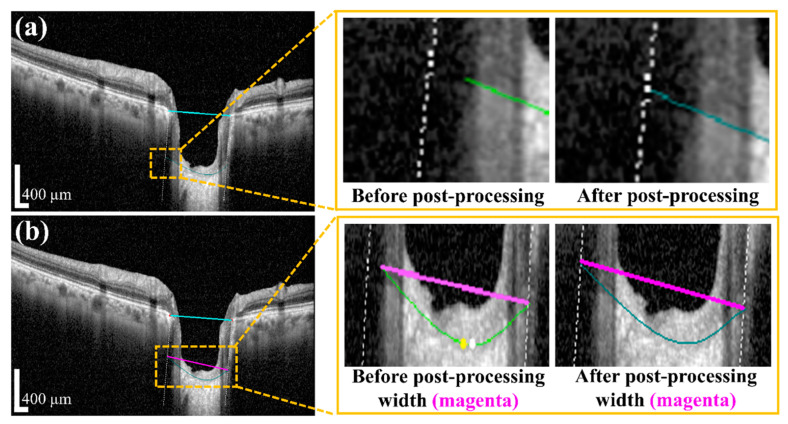
The results of post-processing: (**a**) Post-processing results of anterior LC curve (inside the box indicated the before and after post-processing of the anterior LC curve); (**b**) calculation of post-processing result of the width. Scale bar: 400 µm.

**Figure 11:**
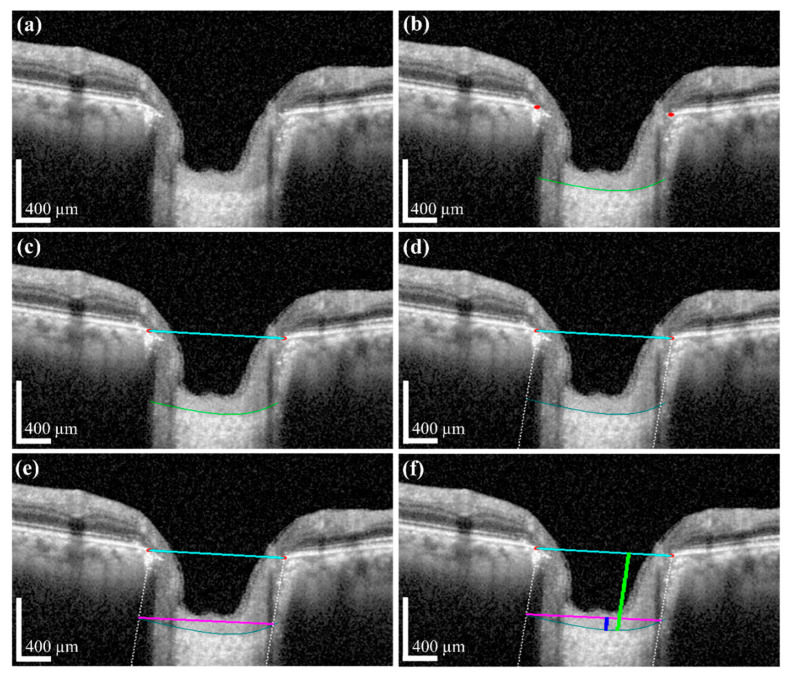
Results of each image processing step for quantification: (**a**) Original OCT image for investigation. (**b**) Proposed deep learning model and post-processing are performed to obtain the LC boundary curve of the BMO points. (**c**) Acquired information after post-processing and two BMO points (red dots) were connected to create a BMO reference line (cyan color line). (**d**,**e**) A vertical dotted line is lowered at two BMO points, and the points meet with the two ends of the anterior LC curve (teal color curve) and create a new line to connect with the two ends of the anterior LC curve (magenta color line). (**f**) The line drawn perpendicular to the anterior LC curve and the BMO baseline is called *LCD* (lime green color line), and the straight blue color line drawn vertically between the width (w) and anterior LC curve is called *LCCD*. Scale bar: 400 µm.

The quantification results acquired by BMO and the anterior LC curve with its parameters can indicate the shape of the post-processing and the anterior LC surface. After successfully applying the post-processing method, we determined the *LCD* and *LCCD*, and *LCCI* information. Figure 10a shows the results of interpolating the area covered by the blood vessel shadow using a polynomial regression curve obtained via the post-processing operation. It can be seen that the vertical white dotted line from the BMO points is not connected to the anterior LC curve before processing, but after post-processing, the white dotted line fitted the anterior LC curve. After the termination of processing, the width (*w*) was connected as a new line from the two end points of the anterior LC curve. Figure 10b exhibits the width (*w*) of the anterior LC surface curve before and after post-processing. In the quantification step, each operation of the quantification process was shown in Figure 11: the original image used for the visualization for post-processing was shown in Figure 11a, while Figure 11b presents the BMO points (red dots) and anterior LC surface curve (teal-colored curve) information, which was obtained from the proposed DL model. The resulting generated BMO reference line (the cyan colored line) is connected by two BMO points carried out after the post-processing operation, as shown in Figure 11c. The vertical dotted line was lowered to meet with the two ends of the anterior LC surface curve and create a new line connected to both terminals of the anterior LC surface curve (the width (*w*)), as shown in Figure 11d,e, respectively. Finally, Figure 11f depicts the results of quantification after the calculation of each morphological parameter.

#### 3.2.4. Statistical and Case Analysis

**Figure 12:**
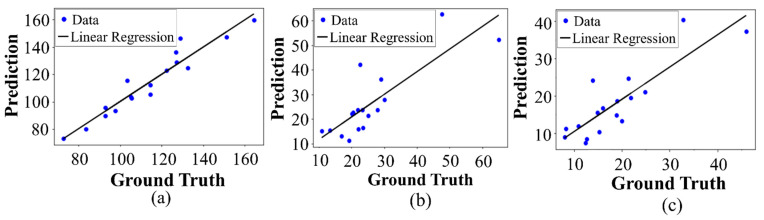
Correlation between the predicted vs. ground-truth values: (**a**) The correlation between prediction and ground-truth for *LCD* was around R² = 0.9649. (**b**) The correlation between prediction and ground-truth for *LCCD* was around R² = 0.6112. (**c**) The correlation between prediction and ground-truth for *LCCI* was around R² = 0.7015.

The quantification method was verified by statistically comparing the performance results generated from the predicted and ground-truth values. This verification was done by linear regression analysis of the acquired values. Significant quantification results of the acquired parameters of the proposed system will show a strong positive correlation between the predicted and corresponding ground-truth values and is verified by using the coefficient of determination: its value will be close to 1 when the residual of two values is small. After the linear regression analysis of the ground-truth and predicted values, the correlation between them is shown in Figure 12, in which we can see that the correlation was very large and the coefficient of determination for each parameter was close to 1.

**Figure 13:**
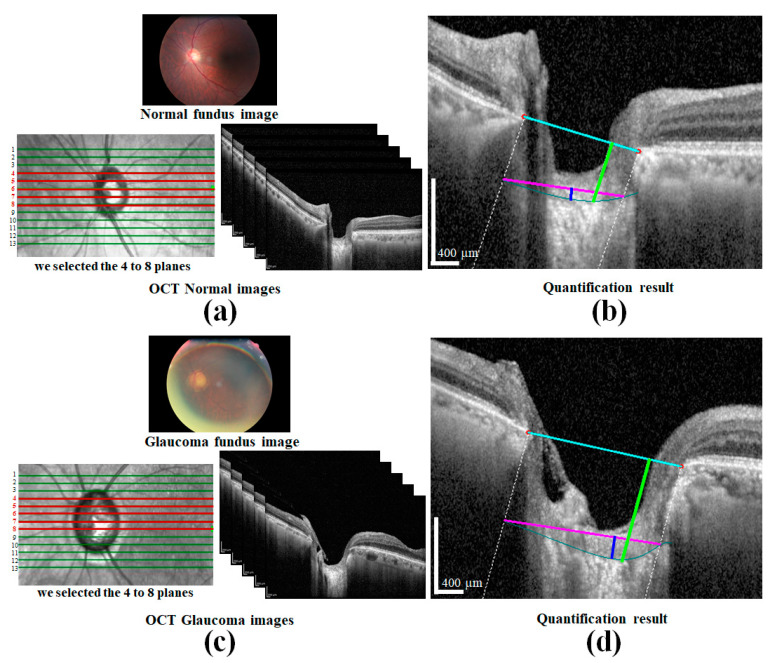
Comparison of normal and glaucoma eye images: (**a**) A normal fundus image and an OCT image after the sampling operation; (**b**) the corresponding quantification results for the normal OCT image (cyan line is the BMO reference line, teal color curve is anterior LC curve, lime green color vertical line is *LCD*, magenta color line is width (w), and blue color line is *LCCD.* (**c**) A glaucoma fundus image and an OCT image after the sampling operation; (**d**) the corresponding quantification results for the glaucoma OCT image (cyan line denotes BMO reference line, teal color curve is anterior LC curve, lime green color vertical line is *LCD*, magenta color line is width (w), and blue color line denotes the *LCCD*. Scale bar: 400 µm.

To verify our approach, we compared the healthy and glaucoma eyes to detect significant changes in the morphological parameters and their numerical values. A patient with one glaucoma and one healthy/normal eye was used for this comparison. Figure 13a,c presents the fundus and OCT images of the glaucoma and healthy eyes, while Figure 13b,d presents the corresponding quantification results for each image, respectively. The comparison results for each eye are reported in Table 4. According to the statistical data from the table, the respective average parameter values for the healthy and glaucoma eye are as follows: 397.85 and 629.56 μm for the *LCD* and 39.98 and 147.29 μm for the *LCCD*, respectively. After computing the *LCCI* by applying (1), the healthy and glaucoma eye values were 2.92 and 6.9, respectively. From the comparison results, it can be confirmed that the parameter values for the glaucoma eye appear higher than for the healthy eye.

## 4. Discussion and Conclusions

We proposed the DL decision making system that can help doctors automatically quantify the morphological parameters of the anterior LC curve for the diagnosis of glaucoma. In previous work, automatic quantification using image analysis and investigations was conducted by considering the features directly observed by humans with machine learning and different computer vision algorithms. However, recent developments in DL have enabled end-to-end learning with high performance for the effective measurement of human eye problems.

By considering the advantages of a DL methodology, we developed a two-stage DL-based quantification algorithm for analyzing OCT images. The developed system primarily consists of two sections: a two-stage DL model, and a quantization algorithm. The DL model comprises object detection and segmentation models that make the system intelligent and efficient. A quantization algorithm is applied for the computation of each LC parameter acquired from the DL models. The acquired results indicate that our proposed method works very well on images obtained via SD-OCT. Automatic quantification of the anterior the LC surface is technically challenging owing to multiple factors, such as selection of bounding boxes for the detection model, the presence of large BVSs due to the local weak signal intensity, the complex shape of the LC, and defects in the LC leading to detection and segmentation errors. We used the LabelImg tool for the proper selection of target objects and implemented the detection model with YOLOv3 to prepare the dataset for the detection to create bounding boxes. The proposed detection models’ performance for object detection was very effective at overcoming the conventional detection problems. Moreover, we proposed the vessel shadow augmentation technique to minimize the blood vessel shadow errors due to weak signal intensity.

Among the parameters for the system, the anterior LC curve was separately subjected to a post-processing step. The cause behind this was the acquired vertical dotted line between two BMO points becoming lost and disconnected from the two ends of the LC curve. The anterior LC curve was modeled via polynomial regression. The Attention U-net algorithm was used to establish the BMO points and the anterior LC curve information processing. Finally, an image processing algorithm was applied to obtain the numerical values for the *LCD*, *LCCD*, and *LCCI* in the quantification step. 

The detection performances of the proposed object detection model for the BMO and LC were 99.92% and 99.18%, respectively. The predicted and ground-truth values of the proposed DL model for each parameter were compared with a distance-based evaluation matrix index in the segmentation step. For the BMO, the average distances of L1 and L2 were 39.23 and 30.95 µm, respectively. For the anterior LC curve surface, the average difference was 8.35 µm and the average Hausdorff distance was 92.89 µm. Statistical analysis revealed a high coefficient of determination values between the predicted and ground-truth values, thereby confirming that the quantification values obtained were significant.

Big data research on the relationship between glaucoma and morphological parameters reveals that the proposed method can contribute to obtaining high accuracy detection results for diagnosing glaucoma. Moreover, the data currently used were obtained from a single OCT machine in a limited environment. In the future, when using data from various environments, we anticipate that higher-performance models can be built, and more advanced methods of quantification can be developed.

## Figures and Tables

**Table 1:** Calculations for the values of L1 and L2.

Classes	Mean L1 Distance (μm)	Std L1 Distance (μm)	Mean L2 Distance (μm)	Std L2 Distance (μm)
Left BMO	49.87	47.06	38.79	34.31
Right BMO	28.58	34.01	24.13	25.11
BMO	39.23	40.54	31.46	30.95

**Table 2:** The anterior LC curve prediction by applying the segmentation algorithm.

Class	Mean y-Difference (μm)	Std y-Difference (μm)	Mean Hausdorff (μm)	Std Hausdorff (μm)
Anterior LC curve	8.35	3.73	92.89	78.74

**Table 3:** BMO class processing results from the segmentation algorithm.

Class	Accuracy (%)	F1 Score (%)	Dice Coefficient (%)
BMO	0.977807	0.988722	0.247702

**Table 4:** Comparison of the quantification of parameters in the normal and glaucoma eyes.

	LCD (μm)		LCCD (μm)		LCCI (μm)	
Plane Numbers	Normal	Glaucoma	Normal	Glaucoma	Normal	Glaucoma
4	405.72	635.73	23.53	178.44	1.39	7.74
5	411.32	566.47	43.14	111.39	4.48	4.90
6	376.99	598.38	52.49	124.23	3.13	7.41
7	392.13	674.24	33.77	127.83	2.11	5.40
8	403.07	672.96	47.06	194.51	3.49	9.12
Average	397.85	629.56	39.98	147.29	2.92	6.91

## Data Availability

The patient data presented in this study are available on request from the corresponding authors. The data are not publicly available due to privacy.
